# Follow-up care needs and motivational factors for childhood cancer survivors and their parents in Germany

**DOI:** 10.1038/s41598-024-84156-y

**Published:** 2025-01-06

**Authors:** Aleshchenko Ekaterina, Langer Thorsten, Calaminus Gabriele, Glogner Juliane, Hellwig Kathrin, Trocchi Pietro, Swart Enno, Baust Katja

**Affiliations:** 1 Institute of Social Medicine and Health Systems Research, Faculty of Medicine, Otto-von-Guericke University, Magdeburg, Germany; 2 University Hospital Schleswig-Holstein, Campus Lübeck, Lübeck, Germany; 3 Department of Pediatric Hematology and Oncology, University Hospital Bonn, Bonn, Germany

**Keywords:** Pediatric cancer, Cancer survivorship, Follow-up care needs, Theory of planned behavior, Stereotype priming model, Paediatric cancer, Health services, Paediatrics, Patient education, Public health

## Abstract

This study aims to explore the long-term follow-up needs and motivations of childhood and adolescent cancer survivors and their parents to attend follow-up care in Germany, given the inconsistent adherence to national follow-up guidelines. We developed interview guidelines based on the Theory of Planned Behavior and the stereotype priming model to explore motivations and barriers related to follow-up care. We conducted a total of 36 episodic narrative interviews with adolescent (ages 13–17) and adult (ages 18–45) survivors of pediatric cancer, as well as their parents. We analyzed the transcripts qualitatively using thematic content analysis, while quantitative analysis through multiple regression models was used to support the qualitative findings and identify predictors of follow-up care attendance. We identified key themes across age groups, including a strong need to “return to normal life” and a desire for ongoing organizational and social support. Both survivors and parents highlighted specific needs, including timely, personalized health information and practical help with healthcare logistics. Adolescents particularly valued emotional support from their social circles, whereas adult survivors and parents expressed a need for more structured psychosocial and logistical assistance. The findings suggest that both age and the time elapsed since diagnosis play a role in affecting survivors’ perceived control to attend guideline-based follow-up care. Our findings suggest that a differentiated approach to follow-up care, with age-appropriate support structures, and tailored guidelines, may improve adherence among survivors and their parents. To enhance follow-up care adherence among cancer survivors, healthcare providers could offer tailored, age-specific information and practical assistance with healthcare logistics. Additionally, providing emotional and psychosocial support resources for both survivors and their parents can help address their unique needs at different stages of recovery.

***Trial registration***: Registered at German Clinical Trial Register (ID DRKS00025960 and DRKS00026092).

**Supplementary Information:**

The online version contains supplementary material available at 10.1038/s41598-024-84156-y.

## Background

Consistent participation in (long-term) follow-up care following childhood and adolescent cancer is crucial to mitigate the risk of encountering late effects that can manifest years after the completion of treatment^1,2^. Approximately two-thirds of childhood cancer survivors between the ages of 5 and 19 experience at least one chronic health condition, by the age of 45 years its prevalence is close to 90%^3,4^. These conditions include physical, psychological, and social effects^1^. Although research frequently examines distinct aspects of pediatric cancer’s impact by investigating the presence and importance of particular health issues, in reality, its effects are highly interconnected and can be experienced across all areas of life simultaneously, requiring complex follow-up care^5^.

In recent years, several university clinics in Germany have established multidisciplinary long-term follow-up clinics, offering specialized, risk-adapted monitoring for pediatric cancer survivors^6,7^. Additionally, currently special long-term follow-up guidelines are being developed. Despite the recommendation for survivors to adhere to follow-up recommendations, only a limited number of them benefit from it due to various reasons with adherence decreasing after protocol-based follow-up^8,9^. These reasons include external influences, - such as the limited capacity of specialized (long-term) follow-up facilities^10^ or insufficient awareness about the necessity of (long-term) follow-up and (long-term) follow-up options available^11–13^, - and internal factors, linked to the health behavior of survivors and their parents^4,14^.

The Theory of Planned Behavior (TPB), a commonly used framework for understanding health behavior, examines internal motivations to exercise exact types of it through three interrelated perspectives: attitude, subjective norm, and perceived behavioral control^15^. Attitude comprises individuals’ assessments of the positive and negative outcomes of a behavior. Subjective norm encompasses the perceived social pressure from others to engage in the behavior, such as the belief that parents anticipate and desire their attendance at follow-up care. Perceived control reflects a person’s capability to carry out the behavior, encompassing the actual ability to perform the behavior and the individual’s confidence. Unconsciously, health behavior might be affected by broader social and environmental stimuli – “primes” according to Bargh et al.^16,17^. Internal motivations shape the need for follow-up care and affect an intention to attend follow-up appointments.

To identify determinants of (long-term) follow-up care adherence more precisely, we pursue the following goals in this study: (1) to investigate, whether the current (long-term) follow-up care aligns with the actual needs of survivors after cancer in childhood and adolescence and their parents; (2) to assess, if survivors and their parents can effectively navigate (long-term) follow-up care with the existing sources of information available to them; (3) to examine potential factors influencing adherence to (long-term) follow-up recommendations from the survivors´ and their parents’ perspective.

This study is a part of the broader VersKiK project, which aims to investigate the long-term impacts of cancer in childhood and adolescence, adherence to specific (long-term) follow-up guidelines, and (long-term) follow-up needs of survivors and their parents. The overall study design of VersKiK and a detailed description of the design of this study are presented elsewhere^18,19^.

## Methods

### Design

To explore the motivations and needs of survivors and their parents, we developed an episodic narrative interview^20^ guidelines in German language based on the theory of planned behavior^15,21^ including elements of the stereotype priming model in the form of free associations to the terms “cancer”, “follow-up” and “survival”^17^. The type of interview we chose was intended to allow participants to share their experiences in detail and to encourage them to express their needs freely, without any limitations^20^. The detailed development of the interview guidelines is described elsewhere^22^. Interview guidelines are presented in Supplements 1–3. We conducted interviews following the COREQ Guidelines^23^ and Declaration of Helsinki^24^.

### Recruitment

We recruited participants by applying a convenience sampling approach^25^ through specialized media (WIR - a German magazine for childhood cancer patients, survivors, and their parents, and social media platforms of the Children Cancer Foundation) and participating hospitals to obtain a heterogeneous field of participants. In this study, we define a survivor as an individual who has been diagnosed with childhood cancer and completed their treatment, regardless of the treatment type, diagnosis, stage, or grade of cancer. The parent interviewees included both parents of interviewed survivors, and others coming from different families, creating a diverse group with various experiences. The only exclusion criteria for survivors were insufficient cognitive function, which might complicate participation in the interview.

### Data collection

EA and JG conducted 36 interviews multicentric – at the University Hospital Schleswig-Holstein and the University Hospital Bonn, where special multidisciplinary long-term follow-up care is already organized, and the University Hospital Magdeburg with no special long-term follow-up facilities, between September 2022 and March 2023. Due to the pandemic situation related to the coronavirus disease (COVID-19), we have conducted one interview as a video call. The duration of interviews varied from 1 to 1,5 h. Written informed consent was obtained from all participants, for minor participants the signature of a parent was also collected.

The general characteristics of interview participants are presented in Table 1.

**Table 1:** General characteristics of interviewees.

	Year of birth	Diagnosis	Year since Diagnoses	Adult patient	Adolescent	Parents	Relapse
1	1996	suprasellar germinoma	6.8	X			N
2	1992	HL	1.7	X			N
3	1993	Rhabdomyosarcoma	18.1	X			N
4	2006	Osteosarcoma	4.3			X	N
5	2006	HL	1.5			X	N
6	2005	Osteosarcoma	2.6			X	N
7	2002	Ewing’s sarcoma	4.0			X	N
8	2003	ALL	6.0			X	N
9	2004	Nephroblastoma	10.4			X	N
10	2001	Neuroblastoma	3.7	X			N
11	1982	ALL	37.6	X			N
12	2000	NET	20.1	X			N
13	2003	Brain tumour	6.7	X			N
14	2000	Brain tumour	20.1	X			Y
15	1988	HL	21.7	X			N
16	1967	Leukaemia	17.8	X			Y
17	2004	Rhabdomyosarcoma	14.2		X		N
18	2006	Neuroblastoma	14.7		X		N
19	2003	Brain tumour	5.6			X	N
20	2003	Brain tumour	11.6			X	N
21	2004	Rhabdomyosarcoma	14.2			X	N
22	2010	ALL	3.7		X		N
23	2008	ALL	2.7		X		N
24	2004	CML	5.7			X	N
25	2008	ALL	2.7			X	N
26	2010	ALL	3.7			X	N

**Diagnosis**: HL - Hodgkin’s lymphoma, NHL - Non-Hodgkin lymphoma, CML - Chronic myeloid leukemia, ALL - Acute lymphoblastic leukemia, NET – Neuroendocrine tumor; Y - yes, N - no.

### Analyses

To generate topics, we analyzed interview transcripts by applying Hsieh and Shannon’s five-step approach to data analysis^26^. These steps included: transforming text into narrative form, identifying units of analysis and themes, establishing coding rules, implementing the coding system across all narrative data and refining it as necessary, and finally, validating and selecting the ultimate dataset. Themes were defined by EA and reviewed by KH and KB. We methodically classified important concepts and identified recurring themes within the narratives through thematic coding. To accurately capture the essence of the messages, we assigned codes based on the recognized themes and subdivided them as necessary.

A guideline-based interview was conducted according to a modular design. Each question block consisted of an introductory question, a Likert scale question specifying it, and a sub-question based on the Likert scale response. Translated interview guidelines are presented in Supplement 1. To study unconscious primes^17^) underlying health behavior, we asked survivors and their parents in the introductory part of the interview to give associations to the terms ‘cancer’, ‘follow-up’, and ‘survival’. Two researchers (EA and PT) independently evaluated the associations given. They assigned them to one of three categories - positive, negative, or neutral, accordingly awarded as 1.5, 0.5, or 1 point (Variable: association factor, Table 3). To identify which dimensions mostly affect the intention of adult survivors and parents to attend follow-up appointments we run multiple regression models for each group of answers (dimensions of perceived control). The relative strength of the predictors was assessed by using standardized beta coefficients and comparing them across dimensions. Due to the small sample size of the population included in the study (*N* = 4), adolescent survivors were excluded from the regression analyses.

## Results

### Qualitative analyses

We analyzed interview transcripts of adult survivors, adolescents, and parents separately. Identified themes are described further and systematized in Table 2.

**Table 2:** Interview themes.

Adult survivors (*N* = 10)	Parents (*N* = 12)	Adolescents (*N* = 4)
Returning to everyday life	Creating normality	Returning to everyday life
Information about the follow-up care process	Information about the follow-up care	Information about follow-up care
Organisational support of the social environment	Strengthening perceived self-competence through prior information from experts	Organisational support of the social environment
Familiarity with the medical environment	–	Familiarity with healthcare professionals
Regular follow-up examinations	Regular (long-term) follow-up appointments provide reassurance	–
Organization of follow-up care	Organization of follow-up care	–
Psychological support for patients and families		Emotional support from the social environment

We identified common themes articulated in three groups of interviewees. This is, first of all, the need to return to “normal life” (survivors) or to create a “normal life” (parents). Adult survivors mentioned that there was a lack of suitable offers to reintegrate back to normality, as their ability to participate in their previous activities might have been greatly reduced. They also faced difficulties in coming back to work or school, thus not being able to hit important milestones in their life. Returning to everyday life is cited as an essential part of leading a healthy life.

*“… My friends*,* so they didn’t understand that when the treatment was finished*,* not everything was over. I am still sick*,* taking pills and everything*,* and maybe I can’t do everything the way I perhaps wanted or still want to. And I don’t dare to do many things as I did before. And*,* well*,* that wasn’t the case. I felt like understanding was missing. So then I broke ties also. So it was also up to me and yeah. That’s why it sucked. And I was very sporty before. That has also changed. As you can see*,* I’m not like that anymore. And that also restricted a lot*,* eventually*,* yes*,* exactly… …”* (Adult survivor, 26 years old; 6,8 years since diagnoses).

Parents generally understand this need and are trying to establish a normal daily routine for their children to create a sense of normality. This includes returning to school, participating in everyday activities such as school trips, and returning to routines that were in place before the illness. The parents have tried to continue their children’s lives as normally as possible. This also includes promoting independence and creating developmental opportunities.

*“… We support her in everything she wants*,* and try to compensate for the after-effects she has due to cancer as best we can. So in a way that she can be*,* yeah*,* normal as well. Like with hearing aids*,* for example……”* (Parent, survivor 18 years old; 14,2 years since diagnoses).

Another common theme discussed by representatives of all three groups is a need for sufficient and timely information on long-term follow-up. Interviewees generally stated that sufficient knowledge transfer on follow-up is lacking: survivors need timely consultations about their current health condition in an understandable form, fertility issues (ideally starting with the diagnostic phase), and availability of follow-up care. Parents would like to be more involved in the follow-up process.

*“… Because there we also attend workshops. Where experts are invited*,* lectures are given*,* and so on. And there I learn the latest news. It’s also the case that*,* regarding the desire to have children and so on*,* I’ve learned the most there*,* not here*,* not here at the fertility clinic*,* where I was also sent sometimes. If there’s anything new*,* it’s definitely through the German Paediatric Cancer Foundation*,* through the seminars I attend there. And if there are sometimes more specific questions*,* which happens time and again because I ‘m involved with the organization of a gymnastics program and engage recurrently with my diagnosis……”* (Adult survivor, 20 years old; 6,7 years since diagnoses).

*“… At that time*,* we researched a lot ourselves and looked at what is possible. …”* (Parent, survivor 18 years old; 14,2 years since diagnoses).

Both adult and adolescent survivors need organizational support from their social environment to integrate (long-term) follow-up care into their routines.

*“… Without my parents*,* I wouldn’t have been able to make it*,* because of the continual traveling. We do live more than an hour away from here”* (Adolescent survivor, 15 years old; 2,7 years since diagnosis).

At the same time, parents articulated a need for strengthening perceived self-competence through tailored expert-based information on (long-term) follow-up care and late effects. Some parents stated that they were not sufficiently informed before the cancer treatment about the type of support they and their children needed. They felt better prepared when they received additional information about (long-term) follow-up measures and options. The information exchange in self-help groups is also considered to be important.

*“… Something like that would be the suggestion I have. That’s what I’m saying*,* the communication on the pee level. That’s the only thing that I was missing the whole time. That I would’ve liked to talk to other parents in the same situation. Because I learned that you can’t talk about this kind of situation with someone who hasn’t gone through it. …”* (Parent, survivor 19 years old; 6,7 years since diagnoses).

Another important topic for survivors is to keep in contact with familiar healthcare institutions and/or healthcare professionals, as it subjectively facilitates open and honest communication and the family feels more secure, that no details of the current state of health are omitted or overlooked. Survivors appreciate having a dedicated contact person who is keeping an oversight on his/her medical history.

*“…I have known the doctors for almost three years now. And with all of them*,* there is a good understanding. Upstairs with the doctor from the intensive care ward*,* for example*,* he was a football fan*,* like me. With him*,* I got along best. …”* (Adolescent survivor, 15 years old; 2,7 years since diagnosis).

The necessity of regular follow-up is acknowledged both by adult survivors and parents to ensure that potential risks for health will be timely recognized. Survivors generally emphasized that the (long-term) follow-up care should be provided by a healthcare professional with specific knowledge of pediatric cancer. For parents such regular (long-term) follow-up appointments also give a feeling of control regarding the child’s health condition.

*“… To the parents*,* I would appeal that follow-up care is important to monitor the health status of the child. And they ought to be disciplined about it. …”* (Parent, survivor 19 years old; 6,7 years since diagnoses).

At the same time, both groups of interviewees have listed several various organizational difficulties that limit the regularity and timeliness of (long-term) follow-up care. Some survivors feel strained to follow up on various late effects, organize medical appointments at different locations, and match those with their daily tasks. They often have to travel a long way to attend (long-term) follow-up care or they lack information on what to consider during (long-term) follow-up care. Some of them also emphasized that (long-term) follow-up care was not available immediately and lacked continuity for a longer period. Parents also mentioned a lack of continuity of the care provided and insufficient organization of appointments by healthcare providers.

*“… I mean*,* sometimes it’s true that I think “Actually*,* I don’t have the time”. Well*,* I mean*,* that is half a day or a whole day that I have to take off. And then I sometimes think: „Yeah*,* actually I should do this and that now”. Or*,* yeah*,* you feel guilty that you didn’t use the day productively. But in the end*,* you know it is important*,* still……”* (Adult survivor, 30 years old; 1,7 years since diagnoses).

Additionally, adult survivors articulated a need for psychosocial support for themselves and their families. Survivors mentioned that the illness has also had a major impact on their family, which did not receive any psychological support afterward. If support was available, it was mostly considered to be not sufficient.

*“… Because it is a mental problem. You’re scared of relapse. You’re scared of the results [of the diagnostic measures]. And that’s why I feel the support from someone who’s mentally*,* let’s say*,* experienced*,* a professional*,* is so important. And the opportunity to talk to someone*,* or also tell them that you need support in that regard/ Those people who sit there*,* they can also look up addresses*,* they can help you or tell you how to do it or what to try. …”* (Adult survivor, 40 years old; 37,6 years since diagnoses).

Similarly, adolescent survivors articulated a need for emotional support from their social environment, including family, friends, and even their pets.

*“… And everyone reached out but also visited me from time to time. So*,* friends got in touch as well*,* that was really /. So*,* when I got sick*,* that went through the roof. I was also /. Donation appeals and so on everywhere*,* so. A lot of people helped.…”* (Adolescent survivor, 15 years old; 2,7 years since diagnosis).

### Quantitative analyses

We evaluated motivations to attend follow-up through qualitative analyses on TPB dimensions. Agreement between the Likert score and association factor (i.e.: if the answer of the interviewees indicates a positive association) was observed in the large majority of adults and carers.

Table 3 shows the standardized beta coefficients that quantify the strength of the association between intention to attend follow-up and several potential predictors. Among adults, the association between predictors and the “perceived control” in managing their health condition was strong for time since diagnosis, and the association factor (beta = 1.01 and 1.02, respectively) showed that perceived control tends to be higher with increasing time since diagnosis and positive associations with relevant terms of cancer. The highest absolute values of the beta coefficient were calculated among adult survivors for the association between “intention” and association factor (2.03), supporting the relevant role that consistent assessment of central terms of cancer plays in intention to attend follow-up appointments (long-term) and attitude toward follow-up care.

**Table 3:** Results from multiple regression models for predictors of intention to attend follow-up: standardized beta coefficients.

	Social Norm	Perceived Control	Attitude	Intention
**Adult**				
Age at diagnosis	− 1.04	0.38	− 0.39	1.53
Time since diagnosis	− 0.37	1.01	− 0.46	0.90
Diagnosis	− 0.48	0.76	− 0.30	1.86
Relapse	0.28	− 0.73	0.41	0.04
F. Up care visit	− 0.66	0.26	0.42	− 0.38
Association factor with relevant terms of cancer	− 0.47	1.02	− 0.38	− 2.03
**Parent**				
Age at diagnosis	–	0.49	− 0.63	0.98
Time since diagnosis	–	0.84	− 1.35	0.43
Diagnosis	–	− 0.48	0.49	− 0.48
F.Up care visit	–	0.42	− 0.98	− 1.16
Association factor with relevant terms of cancer	–	− 0.58	1.40	1.59

## Discussion

The interview material covers a broad age range, (long-term) follow-up phases, and both perspectives from survivors and parents in three different German university hospitals, including one without an established long-term follow-up structure. Having analyzed three groups of transcripts from episodic narrative interviews both qualitatively and quantitively, we were able to explore in-depth motivations to attend (long-term) follow-up appointments and articulated the care needs of adolescent and adult survivors as well as their parents.

### Motivation to attend (long-term) follow-up

As interview guidelines in this study include elements of both the TPB and stereotype priming model, we have added to existing evidence (e.g^4^). that intention to attend (long-term) follow-up appointments and attitude towards follow-up care is also affected by unconscious preferences in adult survivors and parents. Similarly, previous research has shown that survivors whose parents believed that (long-term) follow-up is useful were more likely to attend follow-up appointments^27^. Similar to findings by Baenziger et al.^4^, we found out that survivors with higher perceived control are more likely to attend (long-term) follow-up appointments. We also identified that among adult survivors the longer the interval from the end of acute treatment, the higher the perceived control over one’s health. A positive internal attitude towards (long-term) follow-up might explain a growing intention to attend (long-term) follow-up with increasing time intervals since treatment. These results correspond to a similar study exploring the possibility of TBH to predict (long-term) follow-up attendance^14^.

### Needs of survivors and their parents

A central need articulated by all groups of interviewees is returning to “normal”, everyday life. As stated in a study conducted by Peikert et al.^28^, cancer in childhood and adolescence affects the whole family system leading to significant changes in everyday life. These changes might positively or negatively affect relationships within a family as well as the daily routines of family members, such as participation in a community’s social life or parents’ working schedules. Adolescent survivors who participated in our study described the role of getting back to school twofold – as a “must” for a feeling of “normality” and reconnecting to their peers, but at the same time as a big challenge due to various reasons. Similarly, a meta-analysis conducted by Wang et al.^29^ has shown that school re-entry is often connected to physical, psychological, and social challenges.

Similar to insights provided by Hendriks et al.^30^, another commonly articulated need is a need for timely and diagnoses-tailored information concerning potential late effects, a (long-term) follow-up “roadmap” and guidance on returning to school, work, or studies. Consequently, limited possibilities to attend a specialized multidisciplinary (long-term) follow-up healthcare facility require constant social environment support in navigating and managing (long-term) follow-up care. Parents also expressed a lack of understandable information and comprehension regarding the overall survivors’ status, as well as a deficiency in available resources to support them in return to normal life directly after follow-up has started. As suggested by Bhatt et al.^31^, timely information could enhance an individual’s resilience by improving psychological readiness and decreasing survivors’ feelings of loss. Additionally, the need for information on specialized (long-term) follow-up care providers was also expressed by adult interviewees.

Similar to the results shown by Bossert et al.^32^, adult interviewees in our study also mentioned that barriers to (long-term) follow-up care are often related to costs, - as some examinations and appointments are not covered by insurance companies, - and appointment logistics, thus there is a need for organization of care improvement.

Another interconnected need similarly expressed in these two groups of interviewees, is the regularity of (long-term) follow-up appointments with healthcare professionals with specialized knowledge on pediatric cancer and its late effects. Similarly to the results of Hendriks et al.^30^, adult survivors and parents are interested in getting multidisciplinary (long-term) follow-up consultations with the ability to have all necessary appointments at the same time at one location. In contrast, the majority of adolescent survivors did not express worries about late effects and connected to the necessity to regularly attend (long-term) follow-up. This difference highlights a distinct disparity between the perspectives of adolescent survivors, their older peers, and parents’ views on late effects, similar to the results shown by Petersen et al.^33^. It might be explained by the fact that (long-term) follow-up care is mostly continued by pediatric oncology until the age of 18 even if protocol-based (long-term) follow-up care has been finished. Our study adds to the recent research that both groups of survivors indicate a need to receive long-term (long-term) follow care either within familiar healthcare institutions or supported by familiar healthcare professionals from pediatric oncology.

Survivors, participating in our study, have also articulated a need for psychological support. Interestingly, adult survivors are keener to get it from a healthcare professional, - e.g. psychotherapist or social worker, - while adolescent survivors indicate a need for non-professional emotional support from peers and their actual wider social environment. A study conducted by Petersen et al.^33^ has shown that survivors have a strong motivation to engage in physical activity, particularly if it enables them to reconnect with old friends or establish new friendships. Parents also acknowledged the role of friends as a motivating factor to come back to “normal life” for their children. This indicates a shared opinion between survivors and their parents regarding the significance of social support networks, including both existing and new friendships, for the well-being of survivors shortly after treatment. At the same time, parents serve as emotionally important contact persons, which might limit survivors` drive for autonomy during adolescence and thereby affect age-appropriate social and emotional development. As concluded by Winzig et al., “Survivors long to be understood in what they have been through and consider their parents to understand their perspective best because they had been with them throughout the disease trajectory”^34^ (p. 8).

### Strengths and limitations

The main strength of the present study is the interview guideline’s structure, allowing both qualitative and quantitative analyses, and minimizing a social desirability bias. Also, the foundation of multiple interconnected theories aims to gain a comprehensive understanding of the factors influencing adherence among pediatric cancer survivors and their parents. The involvement of a multidisciplinary researchers’ team with experts from healthcare research both medical and psychological clinical experts is also a strength of this study.

However, the study has several limitations. Firstly, cancer survivors and their parents might have been constrained by sharing negative information and therefore give socially acceptable responses. To mitigate this limitation, we constructed interview guidelines based on different types of questions, including free associations and Likert scale questions. Secondly, we conducted the interviews on a volunteer basis and mostly in hospitals with an established long-term follow-up care structure, thus the answers might not reflect the perspectives of pediatric cancer survivors who are not aware of the late effects of cancer in childhood and adolescence and have no information on (long-term) follow-up care. This also might have affected the answers as survivors and/or their parents might have fear to deteriorate their relationships with (long-term) follow-up healthcare providers through giving “undesirable” answers. Considering the extended time elapsed since diagnosis for some participants, there is a potential for recall bias, especially concerning details and perceptions of follow-up care experiences; this limitation may impact the accuracy of reported adherence patterns and motivations. Finally, survivors and/or their parents might have been not ready to fully share potential traumatic experiences of cancer or still suffer under cancer stigmatization. Due to the relatively small sample sizes of the study population examined, the results of the additional and explorative quantitative analyses can only indicate an approximate trend concerning the assessment of predictors of the survivor’s behavior.

## Conclusion

This study provides valuable insights into the distinct follow-up care needs and motivational factors of childhood and adolescent cancer survivors and their parents. The findings indicate that survivors benefit from a personalized, age-appropriate approach to (long-term) follow-up care, which supports their transition to a “normal” life post-treatment and addresses their unique physical, psychological, and logistical challenges. For adolescent survivors, emotional support from their social environment, including family, friends, and school, is essential for reintegration into everyday life and maintaining adherence to (long-term) follow-up. In contrast, adult survivors and parents require additional guidance on the long-term implications of cancer treatment and practical support to navigate the healthcare system, often facing challenges such as long distances to specialized clinics, time constraints, and fragmented care.

These insights can inform the development of tailored follow-up guidelines that recognize the evolving needs of survivors as they mature. Healthcare providers can use this knowledge to enhance support structures, such as providing regular updates on health and fertility options, fostering familiarity with healthcare professionals, and ensuring access to psychological and logistical resources. Additionally, involving parents in educational and planning sessions can empower them to assist survivors in adhering to (long-term) follow-up care, thereby improving overall health outcomes. By integrating these findings into follow-up practices, healthcare systems can better support survivors and parents in achieving sustainable, long-term adherence to (long-term) follow-up care and ultimately enhance survivors’ quality of life.

## Electronic supplementary material

Below is the link to the electronic supplementary material.

Supplementary Material 1

Supplementary Material 2

Supplementary Material 3

## Data Availability

The anonymised datasets analysed during the current study are available from the corresponding author on reasonable request.
