# Supplementary material for: Follow-up care needs and motivational factors for childhood cancer survivors and their parents in Germany
Source: Sci Rep. 2025 Jan 6;15:972. doi: 10.1038/s41598-024-84156-y (PMC11704210; doi:10.1038/s41598-024-84156-y)

**Interview guideline for adolescents**

1. **attitude**

**PC – perceived control**

**SN – subjective norm**

**I – intention**

**SP – subjective prime**

The interview guideline begins with a task to tell a personal survivorship story, and give free associations with terms “cancer”, “follow-up” and “survivorship”. Further, this interview guideline include 8 main question blocks for adolescents and 4 additional question blocks on transition topics. Each block consists of an introductory question followed by thematically connected sub-questions. A sub-question will be asked based on the answer given to an attitude question (in form of five-point Likert scale – agreement or intensity).

1. A personal survivorship story and free associations (“cancer”, “follow-up”, “survivorship”) ***(SP)***

2. Can you currently lead a live as you wish to (school/training, free time, friends)? ***(SN)***

**Likert:** How much does your cancer still affect your everyday life? (*Intensity scale)*

*If lower half of the scale:* Why, in your opinion, is everything ok in your life?

*If upper half of the scale*: What, in your opinion, might be the reason for things going differently?

3. Are your family members concerned about their own health? How do you see it? ***(SN)***

**Likert:** Please evaluate the extent to which your family members/friends take care of themselves. *(Intensity scale)*

*If lower half of the scale:* Is your intension to attend follow-up appointments affected by it?

*If upper half of the scale*: Do your family members/friends support you in attending follow-up?

4. What do you understand under healthy lifestyle? ***(PC)***

**Likert**: To which extent, do you think, you can contribute yourself to living a healthy life? (*Intensity scale)*

*If lower half of the scale*: Why do you find it difficult to contribute to it? If there is anything that stops you?

*If upper half of the scale*: What exactly do you do to live healthy? Why is it easy for you?

5. In your opinion, how is follow-up organized? ***(A)***

**Likert:** I am satisfied with the follow-up appointments. (*Agreement scale*)

*If lower half of the scale*: What are you not satisfied with?

*If upper half of the scale*: What do you like most?

6. Which thoughts do you have by attending follow-up appointments? ***(I)***

**Likert**: Are you afraid to talk to your doctor? Please evaluate your level of anxiety. (*Intensity scale*)

*If lower half of the scale*: Why are you not afraid?

*If upper half of the scale*: What provokes anxiety during follow-up appointments? If there was an appointment where you were less afraid? Why was that appointment different?

7. How can you see if you are doing well now, compared to period of your disease? ***(PC)***

**Likert:** I'm afraid of getting cancer again. *(Agreement scale)*

*If lower half of the scale:* What gives you confidence that you will stay healthy?

*If upper half of the scale:* Is there anything that helps you to reduce anxiety? If yes, please give some examples.

Does follow-up contribute to it?

With whom are you most likely to talk to about your fears?

8. Have you received any information from your doctor to help you to deal with your follow-up? ***(Information need)***

**Likert:** The information given helps me to understand my follow-up better. *(Agreement scale)*

*If lower half of the scale*: What additional information would you like to have to feel more confident about follow-up issues?

*If upper half of the scale*: From where do you get the most information about follow-up? Please give a few examples.

**Transition questions**

1. What do you understand under the change from child to adult care (transition)? ***(A)***

**Likert:** I am ready for the transition. *(Agreement scale)*

*If lower half of the scale:* Why are you not ready for it?

*If upper half of the scale:*  What tasks do you see for yourself in transition?

**Additional question**: Would you like to decide by yourself, when your transition should begin?

2. Are you comfortable being treated as an adult? ***(A)***

**Likert**: To which extent do you feel involved in your follow-up? (*Intensity scale)*

*If lower half of the scale:* Why don't you feel sufficiently involved it?

*If upper half of the scale:* What makes you feel involved?

3. Who organizes your follow-up appointments? ***(PC)***

**Likert:** I am willing to plan my follow-up by myself (*Agreement scale*)

*If lower half of the scale*: Why is it difficult for you?

*If upper half of the scale*: What makes you confident that you can manage it by yourself?

4. Do you think that you'll still need follow-up when you're an adult? ***(A)***

**Likert:** How confident are you to attend your next follow-up appointment? Please evaluate your level of willingness. *(Intensity scale)*

*If lower half of the scale*: Do you need any support in order to attend follow-up appointments regularly?

*If upper half of the scale*: What or who makes it easier for you to attend regularly follow-up appointments?

5. Is psychosocial support within follow-up important for you? ***(A)***

**Intensity scale**


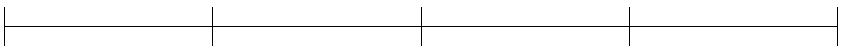


| not at all | a little | moderate | quite | highly |
| --- | --- | --- | --- | --- |
|  |  |  |  |  |

**Agreement scale**

| totally disagree | | disagree | | neither nor | | | agree | | fully agree |
| --- | --- | --- | --- | --- | --- | --- | --- | --- | --- |
|  | |  | |  |  | |  | |  |


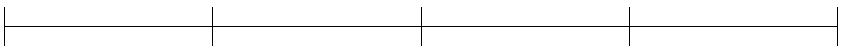

Supplement: Supplementary file 1 — Supplementary Material 1 [file 41598_2024_84156_MOESM1_ESM.docx]
