# Supplementary material for: Follow-up care needs and motivational factors for childhood cancer survivors and their parents in Germany
Source: Sci Rep. 2025 Jan 6;15:972. doi: 10.1038/s41598-024-84156-y (PMC11704210; doi:10.1038/s41598-024-84156-y)
Supplement: Supplementary file 2 — Supplementary Material 2 [file 41598_2024_84156_MOESM2_ESM.docx]

**Interview guideline for parents**

1. **attitude**

**PC – perceived control**

**SN – subjective norm**

**I – intention**

**SP – subjective prime**

The interview guideline begins with a task to tell a personal survivorship story, and give free associations with terms “cancer”, “follow-up” and “survivorship”. Further, this interview guideline include 9 main question blocks for adolescents and 4 additional question blocks on transition topics. Each block consists of an introductory question followed by thematically connected sub-questions. A sub-question will be asked based on the answer given to an attitude question (in form of five-point Likert scale – agreement or intensity).

1. A personal survivorship story of your child and free associations (“cancer”, “follow-up”, “survivorship”) ***(SP)***

2. Does surviving cancer still affect your everyday life and everyday life of your child? ***(SN)***

**Likert:** How much can you influence this impact? *(Intensity scale)*

*If lower half of the scale:* Why do you feel that you cannot influence it? Where do you mostly feel this impact?

*If upper half of the scale*: Why are you confident?

3. Do you / did you feel well prepared to help your child return to a "normal" life after cancer? ***(SN)***

**Likert:** I believe that a "normal" life is possible for my child. *(Agreement scale)*

*If lower half of the scale*: Why do you think that it is rather unlikely? What are potential obstacles?

*If upper half of the scale*: What do you do exactly so that your child can lead a life like other children? Please give some examples.

4. Do you discuss a necessity of follow-up with your child? ***(A)***

**Likert:** I am convinced that follow-up appointments can help my child. *(Agreement scale)*

*If lower half of the scale:* Why are you rather sceptical about follow-up?

*If upper half of the scale*: What do you find most helpful in follow-up appointments?

5. How would you describe an ideal follow-up? ***(A)***

**5a.** **Likert (*in follow-up*):** I am satisfied with the follow-up appointments. (*Agreement scale)*

*If lower half of the scale*: Why are you rather not satisfied?

*If upper half of the scale*: Why are you rather satisfied?

Does your child doctor’s personality contribute to it? Do this doctor’s appointments address your fears?

**5b. Likert** **(*no regular follow-up*):** I would really like an offer like that. (*Agreement scale)*

*If lower half of the scale*: Why would you still not be interested in such an offer?

*If upper half of the scale*: Why do you wish to have such an offer?

6. What are your typical thoughts while making a follow-up appointment for your child? ***(I)***

**Likert:** I find it sometimes difficult to schedule a follow-up appointment for my child (*Agreement scale*)

*If lower half of the scale*: Is there anything you can advise other parents who are struggling?

*If upper half of the scale*: Was there an appointment where it was easier for you? Why was that appointment different?

7. How do you integrate the organization of your child's follow-up into your everyday life? ***(PC)***

**Likert**: How much does planning and attending your child’s follow-up appointments affect your everyday life? *(Intensity scale)*

*If lower half of the scale*: What or who makes it easier for you to integrate the follow-up into your everyday life?

*If upper half of the scale*: Which additional measures or supports of which people and institutions would you need to better integrate follow-up management into your everyday life?

What practical obstacles do you usually have by planning?

8. Is a regular follow-up appointment with the participation of the various specialists useful? Why? ***(A)***

**8a. Likert (*in follow-up*):** I am confident that my child will attend next follow-up appointment or schedule a follow-up appointment soon. *(Agreement scale)*

*If lower half of the scale*: What could help you and your child to attend such an appointment?

*If upper half of the scale*: What makes you confident to attend your child’s next follow-up appointment or to make an appointment soon?

**8b. Likert (*no regular follow-up*):** I would make an appointment if an opportunity will be given. *(Agreement scale)*

*If lower half of the scale*: Why would you still not be interested in such an opportunity?

*If upper half of the scale*: Why do you wish such an opportunity?

9. Have you received any information from your doctor to help you to deal with your follow-up? ***(Information need)***

**Likert:** I consider the information provided to me sufficient. *(Agreement scale)*

**Likert:** I find the information provided to me helpful. *(Agreement scale)*

*If lower half of the scale*: What additional information would you like to have to feel more confident about follow-up issues?

*If upper half of the scale*: From where do you get the most information about follow-up? Please give a few examples.

What helped you the most once the follow-up began?

**Transition questions**

1. What do you understand under change from paediatric to adult care (transition)? ***(A)***

**Likert:** The transition has come/comes at the suitable time for my child (*Agreement scale)*

*If lower half of the scale*: Why not?

*If upper half of the scale*: Why is it/was it a good time?

If a new condition occurs, that you think, might be caused by a cancer, which healthcare professional will you contact? Why?

2. What feelings do you have thinking about your child's transition? ***(A)***

**Likert:** I am willing to give my child responsibility over his/her follow-up. (*Agreement scale)*

*If lower half of the scale*: Why is it difficult for you?

*If upper half of the scale:* How do you encourage your child to take over more responsibility him-/herself?

3**.** What tasks do you see for yourself in the transition of your child? ***(PC)***

**Likert:** The transition time is right. *(Agreement scale)*

*If lower half of the scale*: What might be characteristics of “right timing” for you?

*If upper half of the scale:* What are you confident that your child can manage the transition him-/herself?

4. Is psychosocial support within follow-up important for you? ***(A)***

**Intensity scale**


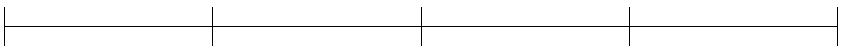


| not at all | a little | moderate | quite | highly |
| --- | --- | --- | --- | --- |
|  |  |  |  |  |

**Agreement scale**


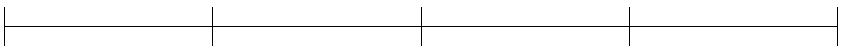


| totally disagree | | disagree | | neither nor | | | agree | | fully agree |
| --- | --- | --- | --- | --- | --- | --- | --- | --- | --- |
|  | |  | |  |  | |  | |  |
