# Supplementary material for: Follow-up care needs and motivational factors for childhood cancer survivors and their parents in Germany
Source: Sci Rep. 2025 Jan 6;15:972. doi: 10.1038/s41598-024-84156-y (PMC11704210; doi:10.1038/s41598-024-84156-y)
Supplement: Supplementary file 3 — Supplementary Material 3 [file 41598_2024_84156_MOESM3_ESM.docx]

**Interview guideline for adults**

1. **attitude**

**PC – perceived control**

**SN – subjective norm**

**I – intention**

**SP – subjective prime**

The interview guideline begins with a task to tell a personal survivorship story, and give free associations with terms “cancer”, “follow-up” and “survivorship”. Further, this interview guideline include 10 main question blocks for adolescents and 4 additional question blocks on transition topics. Each block consists of an introductory question followed by thematically connected sub-questions. A sub-question will be asked based on the answer given to an attitude question (in form of five-point Likert scale – agreement or intensity).

1. A personal survivorship story of your child and free associations (“cancer”, “follow-up”, “survivorship”) ***(SP)***

2. If you had not cancer, would your everyday life be different now? ***(SN)***

**Likert:** How much does your cancer still affect your everyday life? *(Intensity scale)*

*If lower half of the scale:* Why is your life only slightly affected by it?

*If upper half of the scale*: Where do you mostly feel this impact?

3. Are your family members concerned about their own health? How do you see it? ***(SN)***

**Likert:** Please evaluate the extent to which your family members take care of themselves. *(Intensity scale)*

*If lower half of the scale:* Is your intension to attend follow-up appointments affected by it?

*If upper half of the scale*: Do your family members/friends support you in attending follow-up?

4. What do you understand under a healthy lifestyle? ***(PC)***

**Likert:** How much can you contribute yourself to living a healthy life? (*Intensity scale)*

*If lower half of the scale*: Why do you think you can generally make little or no contribution to your health?

*If upper half of the scale*: What exactly do you do to live healthy? Please give some examples.

5. Do you do anything to prevent or reduce specific side effects of your illness or its treatment? ***(PC)***

**Likert:** How afraid are you of getting cancer again? *(Intensity scale)*

*If lower half of the scale:* What gives you confidence that you will never get cancer again?

*If upper half of the scale*: Is there anything that helps you to reduce anxiety? If yes, please give some examples.

Does follow-up contribute to it?

6. What do you feel about follow-up care? Why? ***(A)***

**6a. Likert (in follow-up):** I am satisfied with the follow-up appointments. (*Agreement scale)*

**6b. Likert (no follow-up):** I would really like an offer like that. (*Agreement scale)*

*If lower half of the scale*: With what are you not satisfied?

*If upper half of the scale*: What is most helpful?

7. If you have any conditions, will you bring them up during your follow-up appointment? ***(I)***

**Likert:** I find it sometimes difficult to discuss my conditions with my doctor. (*Agreement scale*)

*If lower half of the scale*: Why is it usually easy for you to discuss it with your doctor?

*If upper half of the scale*: If there was an appointment where it was easier for you? Why was that appointment different?

Does your doctor’s personality contribute to it? Do this doctor’s appointments address your fears?

8. How do you integrate follow-up into your everyday life? ***(PC)***

**Likert**: How much does planning and attending your follow-up appointments affect your everyday life? *(Intensity scale)*

*If lower half of the scale*: What or who makes it easier for you to integrate the follow-up into your everyday life?

*If upper half of the scale*: Which additional measures or supports of which people and institutions would you need to better integrate follow-up management into your everyday life?

What practical obstacles do you usually have by planning?

9. Is a regular follow-up appointment with the participation of the various specialists useful? Why? ***(A)***

**9a. Likert (in a regular follow-up):** I am confident to attend my next follow-up appointment or schedule a follow-up appointment soon. *(Agreement scale)*

*If lower half of the scale*: What could motivate you to attend your next follow-up appointment or schedule a follow-up appointment soon?

*If upper half of the scale*: What makes you confident to attend your next follow-up appointment or to make an appointment soon?

**9b. Likert (no regular follow-up):** I would make an appointment if such an opportunity will be given. *(Agreement scale)*

*If lower half of the scale*: Why would you still not be interested in such an opportunity?

*If upper half of the scale*: Why do you wish such an opportunity?

10. Have you received any information from your doctor to help you to deal with your follow-up? ***(Information need)***

**Likert:** I consider the information provided to me sufficient. *(Agreement scale)*

**Likert:** I find the information provided to me helpful. *(Agreement scale)*

*If lower half of the scale*: What additional information would you like to have to feel more confident about follow-up issues?

*If upper half of the scale*: From where do you get the most information about follow-up? Please give a few examples.

**Transition questions**

1. What was your experience with your change from paediatric to adult care (transition)? ***(A)***

**Likert:** I am satisfied with my transition. *(Agreement scale)*

*If lower half of the scale:* What went wrong? Please give a few examples.

*If upper half of the scale*: What was good about it? Please give a few examples.

2. Has anything changed in your medical care since you switched to adult healthcare? ***(A)***

**Likert:** The transition has brought significant changes to my care. *(Agreement scale)*

*If lower half of the scale:* What helped you get through this process smoothly?

*If upper half of the scale*: In which areas was this change the strongest? Please give a few examples.

3. How would you describe an ideal follow-up? ***(A)***

**Likert:** My family doctor addresses my specific follow-up needs (examinations, doctor appointments). *(Agreement scale)*

*If lower half of the scale:* What difficulties has this caused you? Please give a few examples.

*If upper half of the scale:* How does your family doctor specifically support you?

4. Is psychosocial support within follow-up important for you? ***(A)***

**Intensity scale**


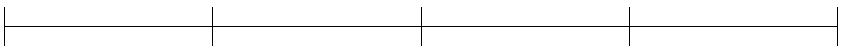


| not at all | a little | moderate | quite | highly |
| --- | --- | --- | --- | --- |
|  |  |  |  |  |

**Agreement scale**


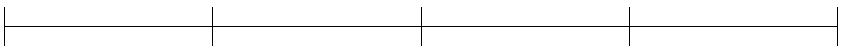


| totally disagree | | disagree | | neither nor | | | agree | | fully agree |
| --- | --- | --- | --- | --- | --- | --- | --- | --- | --- |
|  | |  | |  |  | |  | |  |
